# Variation in public hospital costs for children and young patients from priority populations: An Australian health service economic analysis

**DOI:** 10.1371/journal.pone.0340780

**Published:** 2026-01-22

**Authors:** Rezwanul Rana, Karen Zwi, Jahidur Rahman Khan, Seaneen Wallace, Raghu Lingam, Henry Cutler

**Affiliations:** 1 Macquarie University Centre for the Health Economy and Australian Institute of Health Innovation, Macquarie University, Sydney, Australia; 2 Sydney Children’s Hospitals Network, Sydney, New South Wales, Australia; 3 School of Clinical Medicine, University of New South Wales, Sydney, New South Wales, Australia; Medical Research Council, SOUTH AFRICA

## Abstract

The extent to which inpatient hospital costs for children and young patients (CYP) from the priority population in Australia differ from their peers is unknown. Using a multivariate analysis approach, this study (1) investigated variations in inpatient hospital costs between priority and non-priority CYP; and (2) identified the associations between CYP patient characteristics and inpatient hospital costs. Priority populations were defined as Indigenous Australian, National Disability Insurance Scheme (NDIS) participants and refugee/asylum seeking CYP. They were identified using electronic medical records (EMRs) within the Sydney Children’s Hospitals Network (SCHN). Data from inpatient hospital admissions (public hospitals) by CYP aged 0–17 years between January 2015 and December 2019 within the SCHN were collected from the EMRs. This study employed mixed-effects generalised linear models (GLMMs) with a log-link function and gamma distribution to investigate patient factors associated with inpatient hospital costs. The model incorporated demographic characteristics, socioeconomic and location variables, and hospital admission severity as covariates. Gender (females 1.03 times higher than males), area of residence (1.12 times higher) (non-major city vs. major city) and socioeconomic status (1.04 times higher) (living in socially disadvantaged areas vs. living in less disadvantaged areas) were significantly associated with inpatient hospital costs. Priority CYP who were Indigenous Australian (1.07 times) and NDIS participants (1.02 times) were associated with substantially higher costs compared to those who were non-Indigenous Australian or non-NDIS participants, respectively. Australian CYP with multiple disadvantaged social statuses (priority CYP living in non-major cities) were associated with higher inpatient hospital costs. Reducing health disparities for priority CYP presents a potential opportunity to lower overall hospital inpatient costs in Australia.

## 1. Introduction

Closing the health gap between priority populations (Indigenous Australians, National Disability Insurance Scheme (NDIS) participants, refugees/asylum seekers) and non-priority populations is an Australian Government policy imperative [1,2]. Research on hospital care utilisation across diverse populations has highlighted variations in healthcare access across priority populations. Indigenous Australians who exhibited significantly higher rates of chronic disease and potentially preventable hospitalisations experienced prolonged hospital stays and increased healthcare costs [3]. In contrast, the refugee population in Australia was associated with lower hospitalisation rates for total and acute ambulatory care sensitive conditions compared to the Australian-born population [4]. Children from culturally and linguistically diverse backgrounds constituted a significant risk factor for discharge against medical advice [5].

While studies have examined micro factors related to healthcare costs in specific clinical subpopulations, research on hospitalisation use and costs associated with children and young people (CYP) from priority populations in Australia is limited [4,6–10]. The lack of data on ethnicity and race in Australia poses a threat to achieving health equity and limits the availability of reliable data to inform policy, service delivery, and funding decisions [11]. Hence, further research is needed to investigate the diversity in hospital costs between priority and non-priority CYP, leveraging quality data. While evidence of heterogeneity in health and access to care between priority and non-priority populations in Australia is irrefutable, a critical knowledge gap exists regarding the magnitude of disparity. If admitted, a larger disease burden and limited access to primary care faced by priority CYP may cause a significant increase in hospital costs.

Understanding cost variations in priority population groups can help governments and healthcare system managers evaluate the costs associated with improving health equity. Identifying cost variability can drive the transition towards value-based healthcare, aiding policymakers and providers to develop cost-reduction strategies [12]. These insights empower providers and payers collectively to design reimbursement models that reward cost containment and quality improvement. This study seeks to enhance understanding of factors influencing public hospital inpatient costs for CYP from priority populations, to help guide policy and service strategies, and to address current knowledge gaps.

Clinical and public health research routinely incorporates cost analysis into experimental and epidemiological studies. However, there are several problems in analysing hospital cost data, such as asymmetry of the distribution of the cost data, extensive observations with zero cost, and error variance (cost data) being heteroskedastic [13]. Furthermore, a subset of patients experiencing complications or severe illness may necessitate additional interventions, incurring substantial costs. Prior research frequently lacked the methodological rigour of employing appropriate multivariate regression models to address these complexities [6–9,14,15], reducing the likelihood of producing an unbiased and efficient estimate. Unlike previous studies, this study used a mixed-effect generalised linear model (MEGLM), which is effective in managing non-normal cost data with flexible distributions and covariate effects [13,16] and provides a more robust estimation by incorporating random effects [17]. To the best of our knowledge, this is the first study to integrate the MEGLM approach in analysing hospital cost data for priority CYP in Australia.

This study has leveraged a robust, prospectively collected electronic dataset from the Sydney Children’s Hospitals Network (SCHN) to comprehensively understand healthcare cost disparities and their associations with CYP characteristics in Australia. Employing the MEGLM approach, this study aimed to (1) investigate variations in inpatient hospital costs between priority and non-priority CYP populations and (2) identify the associations between inpatient hospital costs and the characteristics of CYP populations.

## 2. Method

This study analysed electronic medical record (EMR) data collected from public hospital inpatient admissions undertaken within the Sydney Children’s Hospital Network. The data were collected for all CYP patients aged 0–17 years who were admitted to the hospital between January 2015 and December 2019 (inclusive). This study used de-identified, routinely collected electronic medical record data from the SCHN. The SCHN Human Research Ethics Committee (2022/ETH00145) and the Aboriginal Health and Medical Research Council (1920/22) granted ethics approval for this study. This ethics approval includes the waiver of consent to access and analyse de-identified data. Please see the protocol paper for further discussion [18].

Priority populations were defined as Aboriginal & Torres Strait Islander, refugee/asylum seeking, and NDIS participants [see Table 1] and identified based on Australian evidence of inequity and capacity within SCHN’s EMR.

**Table 1:** Identification of the priority population used in this study.

Priority population	Identification criteria
***Aboriginal & Torres Strait Islander***	Patients were classified as Indigenous if they self-identified as Aboriginal, Torres Strait Islander, or both. Patients whose Indigenous status was missing, not stated, or declined, were categorised as ‘unknown.
***Refugee/asylum seeking***	Refugee or asylum seeker status was not routinely collected at SCHN. Therefore, attendance at refugee clinics, recorded in the EMR, was used to identify refugee/asylum seeker CYP, though this data likely underestimates their true numbers.
***National Disability Insurance Scheme (NDIS) participant***	An NDIS participant is a patient formally approved for the scheme, with this status routinely recorded in the EMR. Introduced in Australia in July 2016, the NDIS provides financial support for eligible individuals with significant, permanent disabilities or those requiring early intervention to access supports outlined in their individualised plans; however, not all CYP with disabilities are included.
***Any priority population status***	Any priority population status was applied to hospital presentations for any of the three above priority population groups (Indigenous Australian, Refugee/asylum seeker and NDIS recipient CYP).

To enhance the identification of priority CYP, the “ever rule” was implemented. CYP were categorised as belonging to a priority group if they were identified as such at least once between 2015 and 2019 in inpatient, ED, or outpatient data. For example, a patient identified as Indigenous during any encounter within this timeframe was classified as Indigenous Australian for all subsequent encounters. The authors had no access to information that could identify individual participants during or after data collection. Please see the protocol paper for further discussion [18].

### 2.1. Variable definition

Table 2 defines the variables used in the multivariate analysis. In total, 246,785 paediatric patients had records (number of hospital encounters) of inpatient hospital costs and were included in the statistical analysis. Variables for the regression model were selected a priori based on clinical relevance and data availability. The outcome variable was inpatient hospital costs, measured in Australian dollars (AUD). 4.3% of the patients did not have hospital inpatient cost data and were excluded from the analysis [20]. Residential area (i.e., postcode) remoteness (categorised as “major cities” versus “regional or remote areas”) was represented by the Accessibility and Remoteness Index of Australia (AIRA+) classification [21]. Residential postcodes were used to apply ARIA+ categories to each encounter after they were assigned based on the patient’s residential address upon admission. Residential area socioeconomic status (SES) was created based on the socioeconomic indexes for areas (SEIFA) index of relative socioeconomic disadvantage (IRSD) score [22]. The measure of the admission severity of the CYP was their length of stay, ICU admission (yes or no), number of diagnoses for the episode of care (coded according to International Classification of Diseases (ICD)-10 Australian Modification (AM) codes) and number of procedures undertaken during the episode of care. The assumption was that a higher length of stay, the number of diagnoses and procedures completed, and ICU admission were associated with higher hospital admission severity and inpatient hospital costs [10,12,23].

**Table 2:** Variable definitions.

Variable name	Type	Measurement
Inpatient cost^a^	Continuous	Minimum value $44.29.
Priority population	Binary	Priority population = 1, and non-priority population = 0.
Gender	Binary	Male = 1 and Female = 0.
Age	Count	Age at admission (measured in months)
Area of residence	Binary	Major city = 0 and otherwise (non-major cities) = 1.
Socioeconomic status^b^	Binary	SEIFA IRSD 0–60 = 0 (living in areas of most socioeconomic disadvantage) SEIFA IRSD scores 61 or more = 1 (living in areas of least disadvantage).
Length of stay	Count	Total number of bed-days considering all episodes bundled by transfer, calculated as: (stay end date) minus (stay start date)
ICU admission	Binary	0 = not admitted to the ICU and 1 otherwise
Number of procedures	Count	No 0 value. Ranges from 1 to 50.
Number of diagnoses	Count	No 0 value. Ranges from 1 to 50. Calculated based on the ICD-10 AM codes.
Length of stay	Count	Total length of stay at the hospital
ICU admission	Binary	0 = Yes and 1 = no
Number of diagnoses	Count	Number of diagnoses for each CYP admission (See the S1 Appendix for details)
Number of procedure	Count	Number of procedures completed for each CYP admission
Interaction term 1^c^	Binary	Priority population x area of residence. It was also considered as an “effect modifier” in this study.
Interaction term 2^c^	Binary	Priority population x socioeconomic status. It was also considered as an “effect modifier” in this study.
Interaction term 3^c^	Binary	Indigenous Australian X NDIS participant. It was also considered as an “effect modifier” in this study.
Interaction term 4^c^	Binary	Refugee or asylum seeker X NDIS participants. It was also considered as an “effect modifier” in this study.

^a^Inpatient costs are primarily calculated using the Australian Refined Diagnosis Related Groups (AR-DRGs) system, which groups patients based on similar clinical characteristics and resource use. Each group is assigned a cost weight reflecting its resource intensity. This weight is used to calculate National Weighted Activity Units (NWAUs), which determine hospital funding. The NWAU is multiplied by the National Efficient Price (NEP) set by the Independent Health and Aged Care Pricing Authority (IHACPA) to calculate base funding. Adjustments are made for special cases such as critical care or long stays. The NEP and cost weights are updated annually to reflect changes in costs and practices due to inflation. Rising costs in recruitment, power, and food also impact inpatient costs by increasing funding pressure. See the study of Kardamanidis, Lim [19] for further discussion.

^b^61 is the median value of the SEIFA IRSD score. The percentiles of SEIFA IRSD scores were 8 (10%), 30 (25%), 61 (50%), 87 (75%), and 96 (95%).

^c^The study treated the area of residence, socioeconomic status, and priority population status variables as covariates if they were not significant effect modifiers in the regression analysis.

Note: The severity of admission for the CYP in the model was controlled using the following variables: length of stay, ICU admission, number of procedures, and number of diagnoses. All the variables were significant factors in higher inpatient hospital costs.

### 2.2. CYP in each category

Priority populations constituted 15.5% of the study population (number of encounters); Indigenous Australians accounted for 7%, NDIS beneficiaries comprised 9.6%, and refugees comprised 0.7% (Table 3). Lastly, 1.8% of CYP were common between two priority population subgroups (Indigenous Australian and NDIS participants or Refugee and NDIS participants). 3,954 Indigenous Australians and 448 refugee CYP were also NDIS participants. Table 3 represents the number of CYP in each category for whom inpatient cost data were available.

**Table 3:** Number of CYP in each category.

Population groups	Total number of encounters	% of the total number of encounters
Non-priority CYP	207,742	84.50
Priority CYP	38,018	15.50
Indigenous Australian CYP	17,160	6.99
NDIS participant CYP	23,619	9.64
Refugee CYP	1,733	0.71
Overlapping CYP between any two categories	4,402	1.79

CYP = children and young people.

Note: Numbers only include CYP for whom inpatient hospital cost data were available. The priority population group included Indigenous Australian CYP, NDIS participant CYP and Refugee CYP.

### 2.3. Statistical analysis

Descriptive statistics were based on percentages and frequencies for categorical and continuous variables, means and standard deviations. Proportions were compared using Fisher’s exact test or chi-square test for categorical data. For continuous data, the Welch t-test or Mann-Whitney U test was used if the data were skewed [24]. The means and standard deviations were calculated for inpatient hospital costs.

This study used hospital costs as the outcome variable and priority population as the primary explanatory variable. The study employed the MEGLM for multivariate analyses, which included demographic, socioeconomic, and illness status variables, as well as additional models with relevant interaction terms (Table 2). MEGLM is an effective and flexible framework for analysing hospital cost data, addressing the challenges of non-normal distributions, clustering, heterogeneity, and longitudinal measurements [17,25,26]. The MEGLM approach incorporates both fixed and random effects within the linear factors [13].

#### 2.3.1. Model specification and analysis.

The means and SDs were calculated for inpatient hospital costs. Multiple records for individual CYP, were adjusted for clustering accordingly. The study used mixed-effect generalised linear models (MEGLM) for both univariate and multivariate analyses. In the univariate model, this study used hospital cost as the outcome variable and priority population as the predictor variable.

The simplified form of the model is given below [17] (please see the S1 Appendix for details),


$${hospital\;cost}_{i}=\beta \;{(Priority\;population}_{i})+b\;{(Random\;effect\;varaible}_{i})+\epsilon$$

where all elements concern all macro units; therefore, ${hospital\;cost}_{i}$ is an n-dimensional vector ($n=\;\sum n_{i}$), ${Priority\;population}_{i}$ and ${Random\;effect\;varaible}_{i}$ are the known n × p and n × q matrices of covariates related to the fixed effects and to the random effects, respectively, *β* is the *p*-vector of unknown fixed effects, *b* is the *q*-vector of unobserved and independent random effects, and ϵ represents the vector of unobserved random errors [27].

This study created two separate datasets for the multivariate analysis. First, the analysis excluded the outliers (1%), resulting in 241,001 non-priority patients and 83,700 priority patients. Second, this study calculated a revised priority population including Indigenous Australian, NDIS recipients and refugee CYP population, resulting in 39,183 priority patients. This study performed the statistical analysis with STATA 17.0 (StataCorp, College Station, TX, USA).

In the MEGLM, the association between the linear predictor $\mu_{ij}$ and the observed response $y_{ij}$ can be specified in several ways, depending on the type of response variable [28]. For example


$$g\left(\mu_{ij}\right)=\eta_{ij}$$

The conditional response distribution is from the exponential family and is characterised by the conditional expectation $\mu_{ij}$ as well as a dispersion parameter θ that affects the conditional variance,


$$Var\;\left(y_{ij}|\mu_{ij}\right)=\;\theta V(\mu_{ij})$$

where the variance function $V(\mu_{ij})$ is determined by the chosen distribution [29]. We employed the MEGLM, which allowed us to use the Gamma distribution with the log link function. Let, the linear predictor $\mu_{ij}$ be the amalgamation of fixed and random effects without the residuals.


$$\mu_{ij}=\text{X}\beta +\text{Z}\gamma \;$$

Here, the generic link function is g(.) which relates to the outcome $y_{ij}$ to $\mu_{ij}$. Furthermore,$g^{-\text{1}}=inverse\;link\;function=h(.)$

and $y_{ij}$ is equal to


y=h(μ)+ ε

For a detailed discussion on the MEGLM, its derivation and interpretation, please see the studies of [13,25,29,30,31].

The modelling approach was driven by two key considerations: the hierarchical nature of the data, with patients nested within hospitals, and the specific characteristics (right-skew, non-normal distributions, large outliers) of the distributions of the dependent variable (total inpatient hospitalisation cost). The cost distribution exhibited a right-skew (Skewness = 19 and Kurtosis = 662), with the mean treatment cost standing at $8,325, escalating to $95,263 at the 99th percentile, and some individuals incurring costs surpassing $1,800,000 per admission.

This study used the MEGLM approach because;

1) Generalised linear model has considerable appeal when applied to the regression analysis of cost data. This is primarily due to their flexibility as parametric analysis tools, enabling the specification of various non-normal distributions while allowing for the manipulation of the link function [32].
2) MEGLM offer substantial utility when addressing non-normally distributed data with random effects, offering an enhanced and versatile means of grouping data into clusters [17].
3) MEGLM encompasses an extensive array of random effects, mirroring the well-established structure of linear mixed models. This characteristic endows MEGLM with the versatility akin to traditional linear models, enabling the inclusion of multiple levels of random effects in diverse hierarchical arrangements [25].

**Fig 1:**
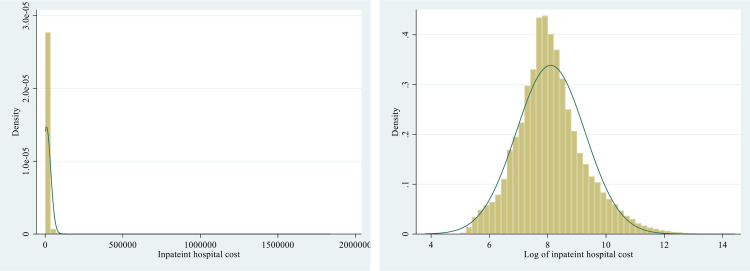
A comparison of the normal and log-normal distribution of the outcome variable (inpatient hospital cost).

Lastly, the primary outcome variable, inpatient hospital costs, was not normally distributed but was naturally log-transformed to bring the distribution closer to normal (Fig 1 and Table 1). Hence, the study used a gamma distribution with a log link function for the multivariate analysis. Past research has also indicated that the Gamma (log link) regression model exhibits favourable performance in estimating population means for healthcare costs across various conditions, and the results demonstrate consistency across different sample sizes [33,34]. Past research has concluded that the gamma distribution is suitable for examining cost-related data [35].

To understand the variation in public hospital costs for CYP from priority populations, this study used seven different MEGLM models. Model 1 included priority population status, with subsequent models incrementally adding key covariates, such as Indigenous status, NDIS participation, and refugee or asylum seeker status, along with their interaction terms. All models controlled for age, gender, area of residence, socioeconomic status, and severity of illness during admission, with standard errors clustered at the patient level.

## 3. Results

Table 4 presents the demographic, socioeconomic, and clinical characteristics of CYP populations with inpatient admissions (2015–2019). Priority CYP admitted to the hospital came from more socioeconomically disadvantaged areas and had a higher mean number of diagnoses and procedures, a longer length of stay, and ICU hours (Table 4). Indigenous Australian CYP were more likely to come from non-major city areas (20%) than the total hospitalised CYP population (10%). Approximately two-thirds of the Indigenous Australian CYP lived in areas of most socioeconomic disadvantage (SEIFA IRSD values less than 61). One in four refugee or asylum seeker CYP, and one in five Indigenous Australian CYP admitted at SCHN, had a respiratory illness. The prevalence of disease in the nervous system was twice as high in NDIS participant CYP (16.2%) as in the non-priority CYP (8.1%).

**Table 4:** Demographic, socioeconomic and clinical characteristics of CYP populations with inpatient admission (2015-2019).

	Non-priority CYP	Priority CYP	Indigenous Australian CYP	NDIS participant CYP	Refugee CYP
N (number of encounters)	207,742	38,018	17,160	23,619	1,733
Gender (male) (%)	58.04	55.2	52.9	55.1	54.1
Age (mean months) [range]	77.9 [0; 460]	84.7 [0; 236]	80.5 [0; 236]	90.8 [0; 223]	88.2 [0; 234]
Resides in major cities (%)	91.1	87.9	82.0	92.3	95.4
Socioeconomic status (disadvantaged areas) (%)	48.2	59.6	65.2	58.9	56.1
Mean length of stay [range]	2.8 [1; 966]	3.7 [1; 667]	3.8 [1; 667]	3.5 [1; 307]	3.7 [1; 123]
Mean no. of procedures [range]	2.9 [1; 50]	3.1 [1; 50]	3.2 [1; 3.2]	3.1 [1; 50]	2.9 [1; 2.9]
ICU Admission [Yes] [%]	4.92	6.1	6.6	5.6	3.1
Mean no. of diagnoses [range] (based on ICD-10 AM codes)	3.1 [3.12; 3.15]	3.9 [3.9;4.0]	3.9 [3.8;4.0]	4.1 [4.0; 4.1]	3.9 [3.7;4.1]
**Prevalence of key diseases and disorders**					
Nervous System (%)	8.1	13.5	10.0	16.2	8.5
Respiratory System	14.5	16.3	19.1	15.4	27.7
Digestive System	12.5	9.9	10.3	9.3	7.5
Musculoskeletal System and Connective Tissue	10.5	9.7	8.1	10.4	7.4
Ear, Nose, Mouth and Throat	8.7	7.9	4.5	7.3	7.9
Kidney and Urinary Tract	4.1	5.7	6.1	6.8	4.8
Blood and Blood Forming Organs and Immunological Disorders	4.5	3.7	3.1	4.3	5.8

CYP = children and young people; ICU = intensive care unit; NDIS = National disability insurance scheme.

Note: The mean number of diagnoses. Conditions that were prevalent in more than 4% of the population were included in the prevalence of the disease and disorders. Detailed results are in the S1 Appendix.

Overall, mean inpatient hospital costs increased by 11.8% and 25.8% from 2015 to 2019 for non-priority CYP and priority CYP, respectively (Table 5). Between 2015 and 2019, the mean inpatient cost of priority CYP populations was consistently higher than that of non-priority CYP populations. The mean difference in inpatient hospital cost between priority and non-priority CYP doubled from 2015 ($1,513) to 2019 ($2,932). The mean inpatient cost showed an increasing trend for all the groups. Lastly, refugee CYP had a lower mean inpatient cost than other priority CYP groups. In addition, refugee CYP exhibited considerable annual cost fluctuation due to small sample sizes (N = 1,733), which increased the impact of singular, expensive admissions on group means and standard deviations.

**Table 5:** Mean inpatient hospital cost (AUD) for CYP over time.

Year	2015	2016	2017	2018	2019
	AUD value				
Non-priority CYP [SD]	7,336.0 [22,095]	7,569.5 [23,201]	7,750.6 [25,085]	8,302.1 [27,440]	8,204.0 [28,243]
Priority CYP [SD]	8,849.7 [27,129]	10,241.7 [36,339]	10,457.7 [35 051]	9,953.6 [28,397]	11,136.1 [43,198]
Indigenous Australian CYP [SD]	9,007.4 [27,690]	9,439.4 [34,136]	11,456.2 [42,816]	9,998.5 [25,390]	11,707.5 [47,875]
NDIS participant CYP [SD]	9,247.3 [30,676]	10,501.1 [36,468]	9,966.7 [28,712]	9,860.8 [30,467]	10,410.5 [37,554]
Refugee CYP [SD]	6,094.6 [10 023]	10,241.0 [26,935]	7,980.7 [14,492]	8,805.1 [20,483]	8,456.1 [20,217]

AUD = Australian dollar; CYP = children and young people; NDIS = National disability insurance scheme; SD = standard deviation.

Note: The number of priority CYP each year was 7,397 in 2015, 8,091 in 2016, 8,565 in 2017, 7,615 in 2018 and 6,350 in 2019.

Multivariate analysis identified priority CYP status, age, gender, and area of residence (living in non-major cities or socially disadvantaged areas) as significantly associated with mean inpatient hospital costs (Table 6). Priority population status was associated with mean hospital costs (Model 1), with CYP in the priority group incurring significantly higher costs (1.04 times [exp.036], 95% CI: 0.01; 0.04) compared to the non-priority group. Hospitalised CYP living in non-major cities had significantly higher (1.12 times, 95% CI: 0.09; 0.12) inpatient hospital costs than those in major cities (Model 1). Hospitalised CYP living in socially disadvantaged areas had 1.04 [exp (.034), 95% CI: 0.05; 0.03] times higher mean costs compared to those from other areas. Female CYP had significantly higher (1.03 times) mean hospitalisation costs compared to male CYP [exp (.029), 95% CI:0.02;0.04].

**Table 6:** Associations between inpatient hospital costs of CYP and their characteristics.

	Model 1	Model 2	Model 3	Model 4	Model 5	Model 6	Model 7
	Coef. (SE)	Coef. (SE)	Coef. (SE)	Coef. (SE)	Coef. (SE)	Coef. (SE)	Coef. (SE)
Priority CYP (Ref: non-Priority CYP)	0.036* (.01)	0.019 (.01)					
Indigenous Australian CYP (Ref: non-Indigenous Australian CYP)			0.053* (.01)			0.052* (.01)	0.056*(.01)
NDIS Participant CYP (Ref: non-NDIS Participant CYP)				0.018* (.01)		0.015 (.01)	0.019 (.01)
Refugee or asylum seeker CYP (Ref: non-Refugee or asylum seeker CYP)					0.021 (0.03)	0.016 (.03)	0.025 (.03)
Age (month)	0.000* (.00)	0.000* (.00)	0.001* (.00)	0.000* (.00)	0.000* (0.00)	0.000* (.00)	0.000* (.00)
Gender (Ref: male)	0.029* (0.00)	0.029* (.00)	0.029* (.00)	0.029* (.00)	0.029* (0.00)	0.029* (.00)	0.029* (.00)
Living in a non-major city area (Ref: major city areas)	0.110* (0.01)	0.101* (.01)	0.108* (.01)	0.112* (.01)	0.112* (0.01)	0.108* (.01)	0.108* (.01)
Living in socially advantaged areas (Ref: Lower socioeconomic status)	−0.034* (0.01)	−0.037* (.01)	−0.034* (.00)	−0.035* (.00)	−0.036* (0.01)	−0.034* (.01)	−0.034* (.01)
Priority CYP x Living in socially advantaged areas		0.023 (0.02)					
Priority CYP x Living in a non-major city area		0.053* (0.02)					
Indigenous Australian x NDIS participant							−0.031 (.28)
Refugee or asylum seeker x NDIS participant							−0.048 (0.7)
**Severity at admission**							
Length of stay	0.06* (0.00)	0.06* (0.00)	0.06* (0.00)	0.06* (0.00)	0.06* (0.00)	0.06* (0.00)	0.06* (0.00)
ICU admission (yes)	0.79* (0.01)	0.79* (0.01)	0.79* (0.01)	0.79* (0.01)	0.79* (0.01)	0.79* (0.01)	0.79* (0.01)
Number of procedures	0.09* (0.00)	0.09* (0.00)	0.09* (0.00)	0.09* (0.00)	0.09* (0.00)	0.09* (0.00)	0.09* (0.00)
Number of diagnoses	0.03* (0.00)	0.03* (0.00)	0.03* (0.00)	0.03* (0.00)	0.03* (0.00)	0.03* (0.00)	0.03* (0.00)
Intercept	7.916	7.918	7.916	7.919	7.919	7.915	7.915
Number of obs (CYP)	179,294	179,294	179,256	179,294	179,294	179,256	179,256
Wald chi2	46,973*	46,973*	46,397*	46,716*	46,021*	47,047*	47,063*

Coef. = coefficient; CYP = children and young people; NDIS= national disability insurance scheme; Obs = observations; SE = standard error;

Note: Demographic variables are age and gender. Interaction term 1 is the place of residence x priority population. Interaction term 2 is the socioeconomic status x priority population.

* = means statistical significance at a 95% confidence interval.

x = means multiplied by Robust standard errors in the parentheses. Std. err. adjusted for clusters (number of groups) based on hash_mrn (medical record number).

The coefficient of the interaction terms also indicated that priority CYP residing in non-major cities (1.05 times, 95% CI: 0.01; 0.09) (Model 2) had significantly higher inpatient hospital costs than priority CYP from major cities. However, the non-significant interaction between priority CYP and living in socially disadvantaged areas implies no moderation effect (Model 2).

The findings also demonstrated that Indigenous Australian status was significantly associated with increased hospital costs (Model 3). For Indigenous Australian CYP, hospital costs were 1.05 [exp (exp.053), 95% CI: 0.04; 0.07] times higher than those of the non-Indigenous Australian CYP populations. The NDIS participant CYP had significantly higher hospital costs (1.02 times; 95% CI: 0.00–0.03) than the non-NDIS participant CYP (Model 4). Refugee CYP had lower mean hospital costs (1.02 times; 95% CI: −0.03; 0.08) than non-refugee CYP, but the result was not statistically significant (Model 5). Model 6 examined the effects of Indigenous background, NDIS participant status, and refugee/asylum seeker status on the outcome variable. Model 7 was built upon Model 6 by incorporating interaction terms. These interaction terms explored how the combined effects of Indigenous background with NDIS participant status and refugee/asylum seeker status with NDIS participant status influence the outcome. Analysis revealed a significant association between Indigenous Australian CYP status and hospital costs in both models (Coef. 0.52, 95% CI: 0.04; 0.07 in Model 6 and Coef. 0.56, 95% CI: 0.04; 0.07 in Model 7). Model 7 suggested that the significant association of the priority population on hospital costs observed in Model 1 was primarily influenced by Indigenous Australian CYP.

## 4. Discussion

The study has identified the relationship between inpatient hospital costs for CYP in the Sydney Children’s Local Health Network, Australia’s largest paediatric hospital network. Priority CYP status was a significant driver of inpatient hospital costs, even after controlling for age, gender, area of residence, and admission severity. Furthermore, the difference in mean inpatient hospital costs (priority vs non-priority CYP) has widened steadily over the past five years at SCHN.

Age, gender, area of residence (non-major city vs. major city), and socioeconomic status (living in socially disadvantaged areas vs. living in less disadvantaged areas) were significantly associated with increased inpatient hospital costs. Priority CYP populations who were Indigenous Australian and NDIS participants showed substantially higher inpatient hospital costs compared to those who were non-Indigenous Australian or non-NDIS participants, respectively. However, hospital costs associated with refugee or asylum seeker CYP did not significantly differ from those of non-refugee or asylum seeker CYP. The mean inpatient costs for refugee CYP were lower than those of the overall priority population group. This likely reflects barriers to accessing required hospital care rather than lower healthcare needs. As hospital EMR data do not capture access-related factors, the observed difference should be interpreted with caution. Nonetheless, prior studies in Australia have shown that refugees face multiple barriers to care, including cultural and language challenges, financial hardship, limited health literacy, and a lack of awareness of available services and healthcare rights [36–39]. Further research is required to understand and compare the healthcare access and cost of refugee CYP with other priority and non-priority CYP in a hospital setting.

Data limitations restrict the ability to definitively explain the reasons behind the higher inpatient hospital costs observed among Indigenous Australian CYP. Nonetheless, this result is consistent with previous studies. Previous research concluded that the elevated risk of chronic conditions among Indigenous Australians led to more extended hospital stays and increased costs [3]. Along with having a higher burden of underlying disease, Indigenous Australians have limited access to early detection and management of common chronic conditions and limited non-hospital alternatives in rural and remote areas [40]. Others found that experiences of racism, prejudice, and discrimination within mainstream healthcare agencies, coupled with the fear of child apprehension, served as potent deterrents to self-reported healthcare access among Indigenous Australians [41,42]. Therefore, an Indigenous CYP may present to hospitals at a later stage of illness with more complex health needs compared to a non-Indigenous CYP. More research is needed to identify and address the underlying factors contributing to this observed disparity in hospital presentations and costs for Indigenous CYP.

The relationship between priority CYP status and hospital costs is multifaceted, arising from a complex interplay of individual, social, and systemic factors. Indigenous Australians have a rich culture that is over 65 thousand years old. The colonisation of Australia saw the destruction of the societal, community and cultural structures that promoted the social and emotional well-being of Indigenous Australians. The ongoing impacts of colonisation have resulted in some Indigenous Australians experiencing social isolation, poverty, unemployment, lack of opportunity in education, and inadequate access to primary healthcare [43,44]. Indigenous Australians living in non-major city areas may have higher costs than Australians living in major cities because of a lack of available care at home or in the community, more severe diseases, differences in hospital discharge practices, systemic barriers, geographical remoteness, and cultural factors [44–46]. Accessible, targeted primary care services for Indigenous Australians are needed to reduce systematic health inequality and may reduce extended stays, ICU hours, and hospital costs [3].

This is the first study to examine the hospital costs of NDIS participants in Australia using the MEGLM approach, while controlling for demographic, socioeconomic, and admission severity variables. The findings showed that NDIS participant CYP had higher inpatient costs than those who were not NDIS participants. The result aligns with the conclusion of past studies, which showed NDIS participants often have functional limitations and higher needs for ongoing healthcare, and functional limitation was significantly associated with increased healthcare use and costs [10,20,47]. However, the SCHN EMR data were not sufficiently detailed to capture the level of functional limitations or referrals for ongoing care. Further research (using linked administrative datasets) is needed to better understand why CYP who were NDIS participants incurred higher inpatient costs than those who were not.

Living in socioeconomically disadvantaged areas was also associated with significantly higher levels of hospital costs for CYP. One study examined how the Australian Refined Diagnosis Related Groups reflected the efficiency and equity of the hospital’s resource allocation in Australia and concluded that patients with lower SES had longer lengths of stay and utilised more inpatient resources [48]. Another Australian study found that children from the most disadvantaged backgrounds are more likely to use public hospital services accessible to patients (without out-of-pocket costs) [49]. However, CYP from socially disadvantaged areas are less likely to use general practitioners, specialists, pathology, and diagnostic imaging services, which are essential for managing chronic health conditions but often require out-of-pocket payments.

Our results also suggested that CYP priority populations from non-major city areas have higher hospital costs than non-priority populations or those living in major cities. Other studies have made similar conclusions [43,44,50]. Rural Australians face poorer health due to poverty, education gaps, and limited healthcare access [51]. Indigenous Australians living in regional areas face an even wider health gap compared to the non-indigenous CYP residing in major cities. A past study found that after controlling for case mix and treatment location, mean hospital costs for Indigenous patients from remote areas were up to 33% higher than for urban non-Indigenous patients [46].

This study has some limitations. The sample size was modest, presenting inherent challenges associated with conducting a retrospective study (e.g., increased susceptibility to bias and inability to draw a conclusion of causation) given that the data were collected prospectively. Post-discharge resource utilisation costs were estimated using EMR data alone, without supplementing with patient interviews to capture care received outside the facility. Furthermore, this study employed broad categorisations for the CYP admitted to hospitals, potentially encompassing a wide range of medical conditions with varying health service requirements. The SEIFA IRSD is an area-based measure, assigning a single score to a geographic area based on population data. This can lead to misclassifying individuals within that area, as their socioeconomic status may not align with the mean score. People living in socially disadvantaged areas may not necessarily be disadvantaged themselves, and vice versa.

## 5. Conclusion

This study revealed differences across multiple dimensions of hospital costs between priority CYP and non-priority CYP in Australia. A widening gap in mean hospitalisation costs between priority and non-priority CYP at SCHN has emerged. This may reflect worsening health gaps, improved access to care, or both. Further research is warranted to address this issue. Despite Australia’s universal healthcare system, costly differences exist in hospital care for CYP with complex health conditions, especially those from Indigenous backgrounds. Hospital costs were also significantly higher for a priority CYP residing in non-major cities and disadvantaged socio-economic areas than those in major cities or advantaged socio-economic regions. Hence, holding multiple disadvantaged social statuses was associated with increased hospitalisation costs. While this may reflect the allocation of greater resources to meet greater needs within the hospital environment, it may also indicate inequities in access to care outside the hospital for disadvantaged populations, such as reduced access to primary care, allied health, and community health programs. To inform public policy, further research is needed to identify the underlying causes and long-term health and economic consequences of hospital cost disparities in CYP inpatient hospital care.

## Supporting information

S1 Appendix This is the S1 Tables Titles. This is the S1 Tables legends.
(DOCX)
