# Supplementary material for: Variation in public hospital costs for children and young patients from priority populations: An Australian health service economic analysis
Source: PLoS One. 2026 Jan 22;21(1):e0340780. doi: 10.1371/journal.pone.0340780 (PMC12826473; doi:10.1371/journal.pone.0340780)
Supplement: S1 Appendix — This is the S1 Tables legends. (DOCX) [file pone.0340780.s001.docx]

**Appendix:**

*Method*

The means and SDs were calculated for inpatient hospital costs. The study used mixed-effect generalised linear models (MEGLM) for both univariate and multivariate analyses. In the univariate model, this study used hospital cost as the outcome variable and priority population as the predictor variable.

The simplified form of the model is given below (Bolker et al., 2009),

$${hospital cost}_{i}=\beta{(Priority population}_{i})+b {(Random effect varaible}_{i})+\epsilon$$

where all elements concern all macro units; therefore, ${hospital cost}_{i}$ is a n-dimensional vector ($n= \sum n_{i}$ ), ${Priority population}_{i}$ and ${Random effect varaible}_{i}$ are the known n × p and n ×q matrices of covariates related to the fixed effects and to the random effects, respectively, *β* is the *p*-vector of unknown fixed effects, *b* is the *q*-vector of unobserved and independent random effects, and $\epsilon$ represents the vector of unobserved random errors (Buscemi and Plaia, 2019).

This study created two separate datasets for the multivariate analysis. First, we excluded the outliers (1%), resulting in 241,001 non-priority patients and 83,700 priority patients. Second, this study calculated a revised priority population including Indigenous Australian, NDIS recipient and refugee CYP population, resulting in 39,183 priority patients. We performed the statistical analysis with STATA 17.0 (StataCorp, College Station, TX, USA).

**Model Specification and Analysis**

In the MEGLM, the association between the linear predictor $\mu_{ij}$ and the observed response $y_{ij}$ can be specified in several ways depending on the type of response variable (Rabe-Hesketh and Skrondal, 2008). For example

$$g\left( \mu_{ij} \right)=\eta_{ij}$$

The conditional response distribution is from the exponential family and is characterised by the conditional expectation $\mu_{ij}$ as well as a dispersion parameter $\theta$ that affects the conditional variance,

$$Var \left( y_{ij} | \mu_{ij} \right)= \theta V(\mu_{ij})$$

where the variance function $V(\mu_{ij})$ is determined by the chosen distribution (Jiang, 2007). We employed the MEGLM, which allowed us to use the Gamma distribution with the log link function. Let, the linear predictor $\mu_{ij}$ be the amalgamation of fixed and random effects without the residuals.

$$\mu_{ij}=X\beta+Z\gamma$$

Here, the generic link function is $g\left( . \right)$ which relates to the outcome $y_{ij}$ to $\mu_{ij}.$Furthermore, $g^{-1}=inverse link function=h(.)$

and $y_{ij}$ is equal to

$$y=h\left( \mu\right)+ \varepsilon$$

For a detailed discussion on the MEGLM, its derivation and interpretation, please see the studies of (Dean and Nielsen, 2007, Gregori et al., 2011a, Manning et al., 2005, Salway and Wakefield, 2008, Jiang, 2007).

**Major Diagnosis Category**

In this study, disease diagnosis was based on the Major Diagnosis Category (MDC) for the Australian National Diagnosis-Related Group (ANDRG). Episodes are assigned to an MDC based on the principal diagnosis. This classification adheres to an Australian standard and is applied uniformly across all hospitals. We did not derive these variables; instead, they were available in the electronic medical records. According to AIHW (2024), the data cube is categorised by the MDC into which the patient’s diagnosis and the associated AR-DRG fall. They correspond generally to the major organ systems of the body. Below is a list of each MDC and the corresponding range of AR-DRGs that fall into each category, as per AR-DRG version 10. For details, please see Australian Institute of Health and Welfare (2024).

**Table A1:** **Major Diagnostic Categories and the AR-DRG ranges covered by each**

| **MDC** | **MDC Description** | **AR-DRG beginning with** |
| --- | --- | --- |
| Pre-MDC | Major procedures where the principal diagnosis may be associated with any MDC | A |
| 01. | Diseases and disorders of the nervous system | B |
| 02. | Diseases and disorders of the eye | C |
| 03. | Diseases and disorders of the ear, nose, mouth and throat | D |
| 04. | Diseases and disorders of the respiratory system | E |
| 05. | Diseases and disorders of the circulatory system | F |
| 06. | Diseases and disorders of the digestive system | G |
| 07. | Diseases and disorders of the hepatobiliary system and pancreas | H |
| 08. | Diseases and disorders of the musculoskeletal system and connective tissue | I |
| 09. | Diseases and disorders of the skin, subcutaneous tissue and breast | J |
| 10. | Endocrine, nutritional and metabolic diseases and disorders | K |
| 11. | Diseases and disorders of the kidney and urinary tract | L |
| 12. | Diseases and disorders of the male reproductive system | M |
| 13. | Diseases and disorders of the female reproductive system | N |
| 14. | Pregnancy, childbirth and the puerperium | O |
| 15. | Newborns and other neonates | P |
| 16. | Diseases and disorders of the blood and blood forming organs and immunological disorders | Q |
| 17. | Neoplastic disorders (haematological and solid neoplasms) | R |
| 18. | Infectious and parasitic diseases | T |
| 19. | Mental diseases and disorders | U |
| 20. | Alcohol/drug use and alcohol/drug induced organic mental disorders | V |
| 21A. | Injuries, Poisoning and Toxic Effects of Drugs: Multiple Trauma | W |
| 21B. | Injuries, Poisoning and Toxic Effects of Drugs | X |
| 22. | Burns | Y |
| 23. | Factors influencing health status and other contacts with health services | Z |

**Table A2 : Prevalent diseases and disorders and associated mean hospital cost based on medical diagnosis codes.**

|  | **N** | **Non-Priority population** |  | **Priority population** |  | **Indigenous Australian** |  | **NDIS recipient** |  | **Refugee** |  |
| --- | --- | --- | --- | --- | --- | --- | --- | --- | --- | --- | --- |
| **Diseases and Disorders** |  | **Freq (%)** | **Mean cost($)** | **Freq (%)** | **Mean cost($)** | **Freq (%)** | **Mean cost($)** | **Freq (%)** | **Mean cost($)** | **Freq (%)** | **Mean cost($)** |
| Nervous System | 18,762 | 8.10 | $7,790 | 13.54 | $9,263 | 10.07 | $10,237 | 16.19 | $9,044 | 8.47 | $6,621 |
| Eye | 5,253 | 2.67 | $4,178 | 1.53 | $4,456 | 1.81 | $4,640 | 1.21 | $4,281 | 0.81 | $5,014 |
| Ear, Nose, Mouth and Throat | 18,123 | 8.69 | $4,601 | 7.99 | $5,926 | 8.47 | $5,732 | 7.25 | $6,262 | 7.99 | $4,594 |
| Respiratory System | 31,289 | 14.54 | $5,414 | 16.33 | $7,124 | 19.10 | $6,290 | 15.40 | $7,614 | 27.71 | $6,787 |
| Circulatory System | 6,000 | 2.98 | $16,630 | 2.14 | 22,413 | 2.66 | $26,023 | 1.58 | $18,137 | 2.57 | $7,642 |
| Digestive System | 25,439 | 12.47 | $5,262 | 9.94 | $7,838 | 10.33 | $7,842 | 9.31 | $8,413 | 7.45 | $18,554 |
| Hepatobiliary System and Pancreas | 1,556 | 0.67 | $30,130 | 1.07 | $26,477 | 1.30 | $26,661 | 0.90 | $26,953 | 2.03 | $22,687 |
| Musculoskeletal System and Connective Tissue | 21,894 | 10.50 | $7,450 | 9.70 | $10,187 | 8.08 | $8,807 | 10.41 | $10,837 | 7.38 | $11,908 |
| Skin, Subcutaneous Tissue and Breast | 7,143 | 3.67 | $3,903 | 1.89 | $4,729 | 2.45 | $4,595 | 1.34 | $5,150 | 1.02 | $11,570 |
| Endocrine, Nutritional and Metabolic | 7,468 | 3.05 | $6,120 | 6.07 | $5,524 | 3.06 | $9,100 | 7.74 | $4,830 | 6.10 | $5,327 |
| Kidney and Urinary Tract | 9,208 | 4.10 | $5,634 | 5.73 | $4,417 | 6.13 | $4,528 | 6.75 | $4,009 | 4.81 | $5,575 |
| Male Reproductive System | 4,599 | 2.38 | $4,256 | 1.14 | $5,220 | 1.17 | $4,397 | 0.98 | $6,005 | 1.08 | $3,715 |
| Female Reproductive System | 785 | 0.38 | $5,853 | 0.35 | $4,771 | 0.36 | $4,302 | 0.39 | $4,688 | 0.27 | $3,301^a^ |
| Newborns and Other Neonates | 5,809 | 2.93 | $31,172 | 1.82 | $56,715 | 2.49 | $39,176 | 1.18 | $86,363 | 0.81 | $7,486 |
| Blood and Blood Forming Organs and Immunological Disorders | 9,309 | 4.55 | $6,134 | 3.69 | $7,327 | 3.05 | $9,831 | 4.26 | $6,003 | 5.76 | $5,600 |
| Neoplastic Disorders (Haematological and Solid Neoplasms) | 8,308 | 3.89 | $14,365 | 4.12 | $16,170 | 5.55 | $17,292 | 2.94 | $16,405 | 3.05 | $11,911 |
| Infectious and Parasitic Diseases | 6,909 | 3.26 | $6,120 | 3.36 | $8,187 | 3.64 | $8,808 | 3.32 | $8,383 | 3.52 | $8,826 |
| Mental Diseases and Disorders | 2,238 | 1.07 | $31,912 | 0.99 | $24,102 | 1.08 | $23,921 | 0.88 | $22,130 | 1.36 | $39,373 |
| Injuries, Poisoning and Toxic Effects of Drugs: Multiple Trauma | 7,685 | 2.67 | $5,307 | 2.32 | $7,063 | 2.78 | $6,317 | 1.87 | $8,507 | 2.37 | $3,499 |
| Burns | 2,119 | 1.05 | $9,406 | 0.79 | $12,979 | 1.46 | $12,108 | 0.23 | $18,627 | 0.20 | $5,319^b^ |
| Factors Influencing Health Status and Other Contacts with Health Services | 12,156 | 5.96 | $3,269 | 4.86 | $8,910 | 4.30 | $8,231 | 5.29 | $9,893 | 4.54 | $5,536 |

^A,b^ means include CYP n<10

**Table A3: Drivers of inpatient hospital costs of CYP**

| **Univariate analysis (mixed-effect GLM)** | **Non-priority CYP** | **Priority CYP** | **Indigenous Australian CYP** | **Refugee CYP** | **NDIS recipient CYP** |
| --- | --- | --- | --- | --- | --- |
| **Distribution model** | **Coef. (SE)** | **Coef. (SE)** | **Coef. (SE)** | **Coef. (SE)** | **Coef. (SE)** |
| Gamma | -0.003 (.01) | 0.21* (.01) | 0.23* (.02) | -0.09 (.05) | 0.18* (.02) |
| Negative binomial | -0.003 (.01) | 0.21* (.01) | 0.23* (.02) | -0.09 (.05) | 0.18* (.02) |
| Number of Observations | 245,760 | 245,760 | 245,719 | 245,760 | 245,760 |
| Number of groups | 122,326 | 122,326 | 122,293 | 122,326 | 122,326 |
| Wald chi2 (Gamma) | 0.14 | 294.58 | 214.40 | 2.69 | 110.51 |
| Wald chi2 (negative binomial) | 0.13 | 294.64 | 214.41 | 2.69 | 110.55 |

Note: Regression results (All population, N= 245,760, Priority N= 88,460)

**Reference**

Australian Institute of Health and Welfare. Australian refined diagnosis-related groups (AR-DRG) data cubes. Australia: AIHW, 2024.
